# Perioperative fatal thrombotic complication after elective meningioma resection in asymptomatic SARS-CoV-2 BA.5.2 (Omicron variant) infection

**DOI:** 10.1016/j.bjao.2023.100131

**Published:** 2023-03-13

**Authors:** Andrea Lavinio, Cinzia Cammarano

**Affiliations:** 1 Neurosciences and Trauma Critical Care Unit, Cambridge University Hospitals NHS Foundation Trust, Cambridge, UK; 2 Department of Anaesthesia, Cambridge University Hospitals NHS Foundation Trust, Cambridge, UK

**Keywords:** elective surgery, neuroanaesthesia, perioperative death, SARS-CoV-2, thrombosis

## Abstract

SARS-CoV-2 infection is associated with hypercoagulability, heparin resistance, and increased perioperative mortality and morbidity. Recommendations on screening and postponement of elective surgery after SARS-CoV-2 infection are being relaxed worldwide. We present a case of fatal thrombotic complication in an asymptomatic incidental SARS-CoV-2 infection (Omicron BA.5.2 variant, first isolated in May 2022) in a triple-vaccinated patient undergoing elective resection of frontal meningioma. The assumption that asymptomatic infection with more recent SARS-CoV-2 variants does not add any perioperative risk remains to be demonstrated. Based on the presented case of unexpected fatal thrombotic perioperative complication in a triple-vaccinated, asymptomatic BA.5.2 SARS-CoV-2 Omicron infection, it would seem prudent to continue to screen for asymptomatic infection and to systematically audit perioperative outcome. Evidence-based perioperative risk stratification of elective surgery in asymptomatic patients with Omicron or future COVID variants relies on reporting of perioperative complications and prospective outcome studies, which would rely on continued systematic preoperative screening.

SARS-CoV-2 infection, whether symptomatic or not, is associated with a three-fold increase in perioperative mortality risk throughout 6 weeks after infection. Viral variants differ in terms of their transmissibility, the severity of illness they cause, and their ability to infect vaccinated patients.^1^ The Omicron SARS-CoV-2 variant, in particular, is characterised by less severe symptoms; increased transmissibility; and the potential to evade immunity acquired through previous SARS-CoV-2 infection, vaccination, or both.^2^

Limited data are currently available on perioperative risk stratification in vaccinated patients with asymptomatic infection by the Omicron variant. Therefore, previous recommendations that, where possible, patients should avoid elective surgery within 7 weeks of SARS-CoV-2 infection remain, unless the benefits of proceeding exceed the risk of waiting.^3^

## Case report

This case report is published with the written consent of the patient's next of kin. A 72-yr-old female with a background of Type 2 diabetes and hypertension treated with metformin and ramipril, respectively, presented with a history of worsening mild left-sided weakness and frequent falls. The patient had also suffered a nondisplaced right ankle fracture approximately 4 months before surgery, which had been treated conservatively and had not restricted her ability to mobilise with the support of a frame. External examination on admission to hospital did not reveal lower-limb swelling, residual pain, or any other signs of deep vein thrombosis.

Brain imaging revealed a right frontal meningioma of approximately 5 cm in diameter with perilesional oedema and mass effect.

The patient was admitted to Addenbrooke's Hospital, Cambridge, UK for elective resection of the tumour. Her neurological status on admission was Glasgow Coma Scale (GCS) 15, with mild left-sided weakness affecting upper and lower limbs. The patient was triple-vaccinated against Covid-19 and was deemed to be immunocompetent, and her admission SARS-CoV-2 polymerase chain reaction (PCR) screening test was negative.

Hospital screening policies have evolved and changed throughout the SARS-CoV-2 pandemic in accordance with WHO directives. At our hospital, routine screening was stopped in September 2022 for all asymptomatic patients admitted for elective and non-elective procedures, unless immunocompromised. As the SARS-CoV-2 screening testing policy had just changed for hospital admission, a SARS-CoV-2 PCR test was performed. This returned a negative result.

Because of significant perilesional oedema, a decision was made to postpone surgery until 6 days after admission for preoperative treatment with dexamethasone 4 mg twice daily. Prophylactic dalteparin 5000 units was administered subcutaneously daily from the day of admission until the evening before surgery. Non-pharmacological prophylaxis consisted of Thrombo-Embolus Deterrent Stockings through hospitalisation and intermittent pneumatic calf compressors during surgery.

On the morning of surgery, preoperative vital signs and blood results were within expected ranges, with the exception of a slightly reduced activated partial thromboplastin time of 23.7 s (reference: 26.7–35.7 s) and prothrombin time of 10.0 s (reference: 10.3–12.9 s). A second SARS-CoV-2 screening sample was taken on the morning of surgery. The result of this second test was still pending at the time of anaesthetic induction, and surgery proceeded in accordance with local policy, which did not require a repeated negative sample.

After uneventful induction of general TIVA, the patient was established on intermittent positive-pressure ventilation in the supine position and the head secured in a Mayfield clamp. Mannitol 10%, 100 ml was administered shortly after induction. Stealth navigation was set up. This was followed by bicoronal scalp incision. The myocutaneous flap was raised. After craniotomy, a frontal bone flap was raised.

Despite stable heart rate and blood pressure, the early intraoperative course was characterised by a significant base deficit (base excess [BE] –11.1 mmol L^−1^ at baseline, after anaesthetic induction) and metabolic acidosis with a mild lactic acidaemia (lactate 2.6 mmol L^−1^) of unclear cause, which improved transitorily and incompletely with fluid resuscitation (4 L crystalloids and 2 units of packed red blood cells). Pulmonary gas exchange remained normal throughout surgery.

The tumour was exposed and debulked uneventfully, and the bone flap was replaced with plates and screws and the scalp closed in layers. Emergence from anaesthesia appeared to be uneventful, and the trachea was extubated approximately 5 h after induction with stable vital signs and neurology (GCS E3V4M6). The base deficit before extubation had reduced to BE –6.0 mmol L^−1^.

Sudden neurological deterioration occurred approximately 15 min after extubation (GCS E1V2M5) with pupils equal and reactive. The patient was re-anaesthetised with propofol and remifentanil, and the airway was secured with maintenance of normal vital signs throughout.

Around this time, the SARS-CoV-2 PCR test that had been sampled in the morning was returned as positive and sequenced as a BA.5.2 2019 nCOV Omicron variant, first isolated May 2022.^2^

Emergency postoperative CT demonstrated unexpected left frontal haemorrhagic infarct, likely to represent a venous infarction, pneumoencephalus, and subdural haematoma and also hypodensities of the posterior circulation.

Under general anaesthesia, the previous incision was reopened and the craniotomy flap raised, revealing a tense brain. The small subdural haematoma was evacuated, and an intraparenchymal intracranial pressure (ICP) monitor was placed to facilitate postoperative management and neuroprotection.

The time trend of plasma fibrinogen indicated an acquired hypercoagulability state, with fibrinogen increasing from normal concentrations (reference range: 1.5–3.3 g L^−1^) preoperatively to 4.2 g L^−1^ postoperatively and peaking at 6.5 g L^−1^ during the neuroscience intensive care unit stay.

**Figure 1:**
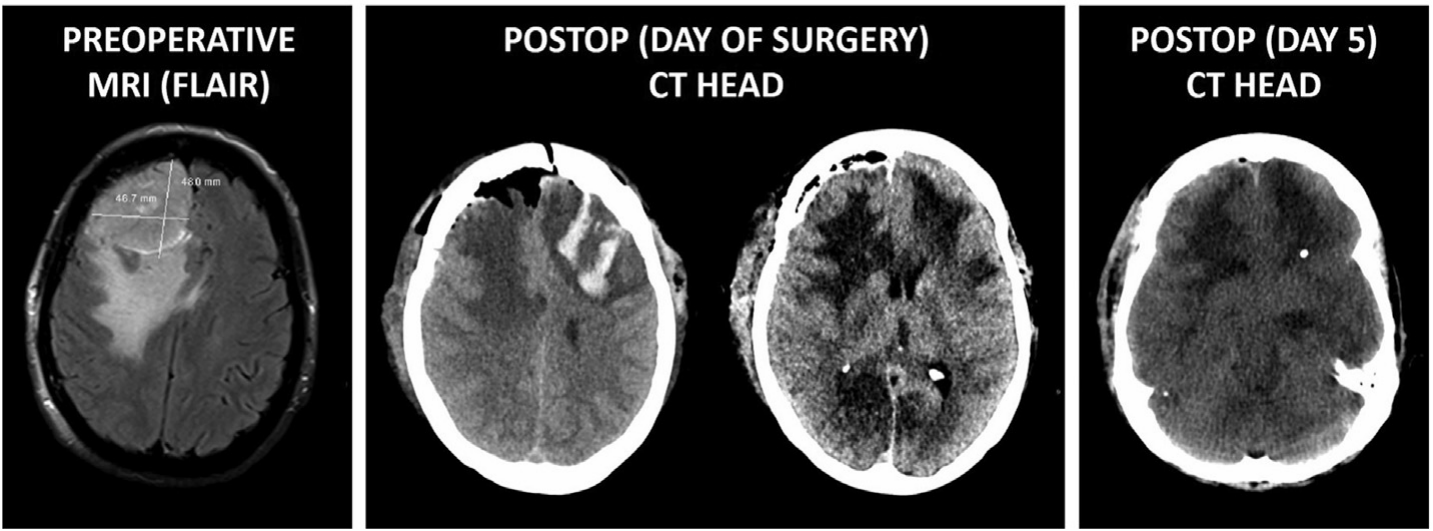
(a) Preoperative MRI: 5 cm right frontal meningioma with associated oedema. (b) Postoperative CT (Day 0): unexpected left frontal haemorrhagic infarct likely to represent venous infarction and new posterior circulation bilateral hypodensities. (c) Postoperative CT (Day 5): evolution of multifocal infarcts with obliterated basal cisterns and sulci, and uncal herniation.

Despite maximal medical treatment, the multifocal intracranial infarcts detected by the CT head performed at admission to neuroscience critical care progressed to hypodense lesions affecting bilateral frontal, occipital, and temporal lobes and of the basal ganglia, with resulting bilateral uncal herniation compressing the midbrain (Fig 1c).

Refractory ICP with ICP >80 mm Hg and diabetes insipidus developed, indicating fatal and irreversible neurological injury. Active care was withdrawn, and death was confirmed on the 12th day after surgery.

## Discussion

We present a case of unexpected fatal venous and arterial thrombotic complications of an asymptomatic incidental SARS-CoV-2 infection (Omicron BA.5.2 variant) in a triple-vaccinated patient undergoing elective resection of a frontal meningioma.

Since the first wave of the pandemic, it became evident that symptomatic SARS-CoV-2 infection was associated with profound hypercoagulability and heparin resistance.^4^^,^^5^ Asymptomatic SARS-CoV-2 infection was also associated with a significant increase in perioperative morbidity and mortality, leading to recommendations to postpone elective surgical procedures for at least 7 weeks after infection.^2^

In view of widespread vaccination, milder symptoms associated with more recent Omicron variants, and the significant backlog of patients awaiting surgery, there might be an opportunity to safely relax such recommendations.

It is important to note that for oncology patients undergoing surgery, requiring expedited surgery, delayed surgical treatment during non-pandemic and pandemic settings has been shown to be associated with worse overall survival when compared with timely surgical treatment.^6^^,^^7^ As such, the balance between the risk of postoperative complications associated with SARS-CoV-2 infection and the risk of worse overall survival associated with delayed surgical treatment should be carefully discussed at a multidisciplinary level and implemented in a patient-shared decision-making pathway.

The near-complete cessation of elective surgery worldwide and the curtailment of all but the most urgent and emergency cases attributable to the SARS-CoV-2 pandemic have burdened health systems for years to come. Barie and colleagues^7^ estimated that the backlog of cases was already more than 28 million in June 2020 worldwide. They suggested that the temptation to sacrifice rigor for speed in resuming elective surgery must be avoided. Crucial elements of a resumption programme include awareness of the status of the pandemic and testing, globally and locally.^7^

Based on proportionate review of medical records and after coronial review, SARS-CoV-2 was included in the medical certificate of cause of death as a ‘causative factor’ (1b) for our patient. This conclusion was based on otherwise unexpected cerebral venous and arterial infarcts leading to a fatal outcome despite adequate pharmacological thromboprophylaxis, and a concomitant increase in fibrinogen concentrations, typically associated with severe COVID-19. A post-mortem was not held.

Whilst this causative link is probabilistic rather than deterministic, it seems reasonable to conclude that the assumption that asymptomatic or mildly symptomatic infection does not add risk remains to be demonstrated.

Evidence-based guidelines to safely restore surgical activity are urgently needed. Based on the present case of unexpected fatal thrombotic perioperative complication in a triple-vaccinated, asymptomatic BA.5.2 n-SARS-CoV2 (Omicron) infection, it would seem prudent to continue perioperative screening for asymptomatic SARS-CoV-2 infection. Whilst it may be safe to perform surgery on asymptomatic or recently recovered patients, robust risk stratification must rely on prospective screening and perioperative outcome monitoring.

## Authors’ contributions

Writing, editing of the case study, discussion and figures, and clinical care: both authors.

## Declarations of interest

The authors declare they have no conflicts of interest.
